# Poor warfarin anticoagulation in long-term thromboprophylaxis: a survey in a southern Croatian county

**DOI:** 10.3325/cmj.2019.60.2

**Published:** 2019-02

**Authors:** Aleksandar Knežević, Marijana Nadinić, Irena Užović Frakin, Vladimir Trkulja

**Affiliations:** 1 Department of Health Studies, Zadar University, Zadar, Croatia; 2 County General Hospital Zadar, Zadar, Croatia; 3 Department of Pharmacology, University of Zagreb School of Medicine, Zagreb, Croatia

## Abstract

**Aim:**

To assess the quality of real-life warfarin anticoagulation in patients requiring chronic thromboprophylaxis in a southern Croatian county.

**Methods:**

We retrospectively analyzed international normalized ratio (INR) values determined over one year (2016-2017) at the Zadar County General Hospital in warfarin-treated patients requiring chronic thromboprophylaxis. The values represent 83.0% of all INRs and were determined in 84.0% of all warfarin-treated patients in the county during the observed period.

**Results:**

Overall 31 162 INRs were taken from 3697 patients, 2240 of whom (20 851 INRs, 3-56 per patient, median 9) were referred with diagnoses requiring chronic thromboprophylaxis: mainly atrial fibrillation/flutter (n = 1508, 14 902 INRs) but also cardiac implants, valvular disease, severe heart failure, and cerebrovascular disease (“other”, n = 732, 5949 INRs). Only 50.1% of all INRs were within the target range, 2.0-3.5, while 43.6% were <2.0, and 6.3% were >3.5. Median crude individual proportion of INRs within the range was 50.0%, while it was 42.0% for INRs <2.0. Only 23.0% of the patients had ≥70% of the INRs within the target range (adequately anticoagulated), while 35.5% had ≤33.3% of the INRs within the range. Conversely, 66.5% of the patients had ≥33.3% INRs <2.0. Adjusted probability of adequate anticoagulation in atrial fibrillation/flutter patients was consistently 25.5% to 27.7%, regardless of the number of determined INRs, while in patients with other conditions it increased from 9.5% to 25.2% with a higher number of INRs.

**Conclusion:**

The achieved level of warfarin anticoagulation in this real-life setting is far below what is needed for effective long-term thromboprophylaxis.

European and American cardiological and neurological societies recommend long-term (life-long) anticoagulation with classical oral anticoagulants (OACs) – vitamin K antagonists (VKAs) – for primary and secondary prevention of stroke/systemic thromboembolism in patients with atrial fibrillation/flutter (AF), rheumatic or non-rheumatic valvular disease, mechanical artificial valves, chronic heart failure and very low ejection fraction (in sinus rhythm), and/or left ventricular thrombi (1-5). Of a number of VKAs that have been introduced into medical practice, the most frequently used worldwide is warfarin. Over the past 10 years, a new generation of orally active anticoagulants has been introduced – new or non-VKA oral anticoagulants (NOACs) – with proven efficacy and safety in primary thromboprophylaxis in patients with non-valvular AF, however warfarin (VKAs) remains the treatment of choice in a number of other settings (1-5). In clinical trials, most of which were conducted in non-valvular AF (the most prevalent condition with increased thromboembolic risk), warfarin reduced the risk of stroke/systemic embolism by around 66% and all-cause mortality by around 25%, without an increase in the risk of major bleedings (1,2). To achieve this balance between the clinical risks and benefits, anticoagulant activity of warfarin, illustrated by the international normalized ratio (INR), should be kept within the range of INR 2.0-3.0, or somewhat higher in patients with mechanical prosthetic valves (depending on their thrombogenic potential) (2,3). Considering the inherently high inter- and intra-individual variability of warfarin anticoagulant activity, partly influenced by genetic determinants but largely by numerous clinically relevant interactions between warfarin and other drugs, foods, and food supplements (2,3,6,7), achieving this goal requires constant frequent monitoring of prothrombin time (INR), particularly at the start of treatment and in patients showing variable INRs (2,3). Guidelines recommend that a patient should spend ≥70% of time with INR within the target range (time in therapeutic range, TTR), as assessed by a method based on linear interpolation of INR values (2,3). This goal is achievable in daily practice (8-10), but this refers to health care systems that incorporate one or more of the measures like wide accessibility of specialized anticoagulation clinics, application of point-of-care devices for determination of prothrombin time at home, use of computer algorithms for dose adjustments, constant determination of TTR, alerting systems for regular INR measurements, and patient registries. However, this goal is commonly not achieved, even in contemporary clinical trials (1,2), and particularly not in daily practice even in developed countries and well-organized health care systems (11,12). TTR, as well as an alternative measure of adequacy of warfarin anticoagulation, the percentage of determined INR values within the target range (12), well predict the success or failure of warfarin thromboprophylaxis: lower values (ie, INR<2.0) predict the increased incidence of thromboembolic events, and predominantly too high or highly variable values predict the incidence of major bleedings (12).

In Croatia, there are no widely accessible coagulation clinics (for the outpatients), no national, regional or institutional patient registries, and the Croatian Health Insurance Fund does not reimburse the use of portable coagulometers. The management of long-term warfarin anticoagulation has been formally assigned to general practitioners (GPs), however the compliance with the regular INR controls and warfarin dose-adjustments are time-consuming and may be difficult to achieve for the outpatients, particularly those with restricted mobility. Previous reports from other settings suggested that warfarin anticoagulation in Croatia was poor (13,14), but to our knowledge there have been no reports providing a deeper insight into the adequacy of warfarin treatment specifically in patients who require long-term anticoagulation. We aimed to assess the adequacy of warfarin anticoagulation in patients requiring long-term thromboprophylaxis in the Zadar County, Croatia, over a one-year period.

## Patients and methods

### Study area and population

Zadar County is a county in the south of Croatia, encompassing a coastal part and several sparsely inhabited islands over a total of 3646 km^2^ (6.4% of Croatian territory). According to the 2011 census (15), the County population is 170 000 (4.1% of Croatian population), 124 400 of whom are older than 19 years (15). They are served by 99 general practices and three hospitals: County General Hospital Zadar (in the city of Zadar, the County center), Special Hospital for Psychiatric Disorders (on the island of Ugljan), and Special Hospital for Orthopedic Surgery (in the town of Biograd) (16). Prothrombin time is determined at the Central Laboratory of the County General Hospital (blood samples can be taken at the Laboratory or at general practices) and at four additional smaller laboratories within a perimeter of 25-60 km.

### Design and ethics

The present study is a retrospective analysis of INR values determined at the Central Laboratory of the County General Hospital over a one-year period between September 1, 2016 and August 31, 2017. It was approved by the institutional Ethics Committee (approval No. 02-6432/18-9/18). During this period, 37 711 INR values in 4377 warfarin-treated patients were determined cumulatively at the five locations in the county. Of those, 31 162 INRs (83%) in 3697 patients (84%) were determined at the County Hospital Central Laboratory. Therefore, the present analysis can be considered a screen of the total county population. Electronic database at the Central Laboratory was searched to anonymously identify all warfarin-treated patients referred for prothrombin time and INR determination. The present study included patients in whom referral International Classification of Disease (ICD) codes indicated that they were under warfarin treatment due to conditions requiring long-term thromboprophylaxis: AF (codes I48, I49), chronic heart failure with low ejection fraction/atrial thrombi (codes I50, I42, also I25), rheumatic or non-rheumatic valve disease (codes I05, I06, I08, I08, I34, I35, I36, I37), history of ischemic stroke or transitory ischemic attack (TIA) (codes I63, I64, I74) (addressed as “advanced cerebrovascular disease”), or because they had prosthetic valves/cardiac implants (codes Z95, Z96).

### Outcomes

For each patient, we determined the proportion of INR values that were within the target (therapeutic) INR range (2.0-3.5), below it (<2.0), or above it (>3.5). In most conditions requiring long-term thromboprophylaxis, of which non-valvular AF is by far predominant, the recommended target INR range is 2.0-3.0 (with 2.5 being the “point-target”) (1-5). We defined the target range as 2.0-3.5 since: a) in some conditions, eg, in patients with prosthetic valves, the recommended INR target is somewhat higher than INR 2.0-3.0, depending on thrombogenic potential of the implanted valves (2,3). Only a minor portion of patients fell into this category and we assumed that most likely they had mechanical prostheses with low thrombogenic potential, hence the targeted INRs would be within the defined range; b) in stably treated patients occasional values >3.0 are often tolerated without a prompt dose adjustment as they could be caused by transient interfering factors (eg, consumption of certain foods, short-term use of non-steroidal anti-inflammatory drugs), and a meaningful increase in the bleeding risk should be expected with INRs >3.5 (2). We defined the “proportion of patients with ≥70% of INR values within the target range” as the primary outcome. The proportion of INRs within the target range correlates well with TTR, hence ≥70% INRs within the range corresponds fairly well to TTR≥70%, which illustrates adequate warfarin-based anticoagulation (12).

### Statistical analysis

Proportion of patients with ≥70% INRs within the target range (adequately anticoagulated) is reported overall and across patient subsets by referral mode (GPs or internal hospital referrals), main diagnosis (AF or “other conditions” cumulatively, since patient numbers by individual conditions were limited), and the number of INRs determined. To evaluate the effects of age, sex, main diagnosis, referral mode, and the number of INRs determined, a modified Poisson regression model with sandwich error estimation was fitted to the proportion of adequately anticoagulated patients. We report adjusted probabilities of adequate anticoagulation and relative risks expressed as prevalence ratios (PR, since we recorded prevalence, not incidence). The number of INRs determined was treated as a continuous variable, and was ln-transformed and geometric-mean centered since interaction terms between indication or referral mode and the number of INRs were included in the model if *P* < 0.1. The effect of increasing the number of determined INRs is expressed as PR by 2-fold increase in the number of INRs. All estimates are given with 95% confidence intervals. For illustrative purposes, patients were also classified as those with 3-7, 8-12, and >12 INRs determined over the observed year, and we additionally report the distribution of patients in respect to the percentage of individual INRs within, below, or above the target range. We used SAS for Windows 9.4 software (SAS Inc., Cary, NC, USA).

## Results

**Figure 1:**
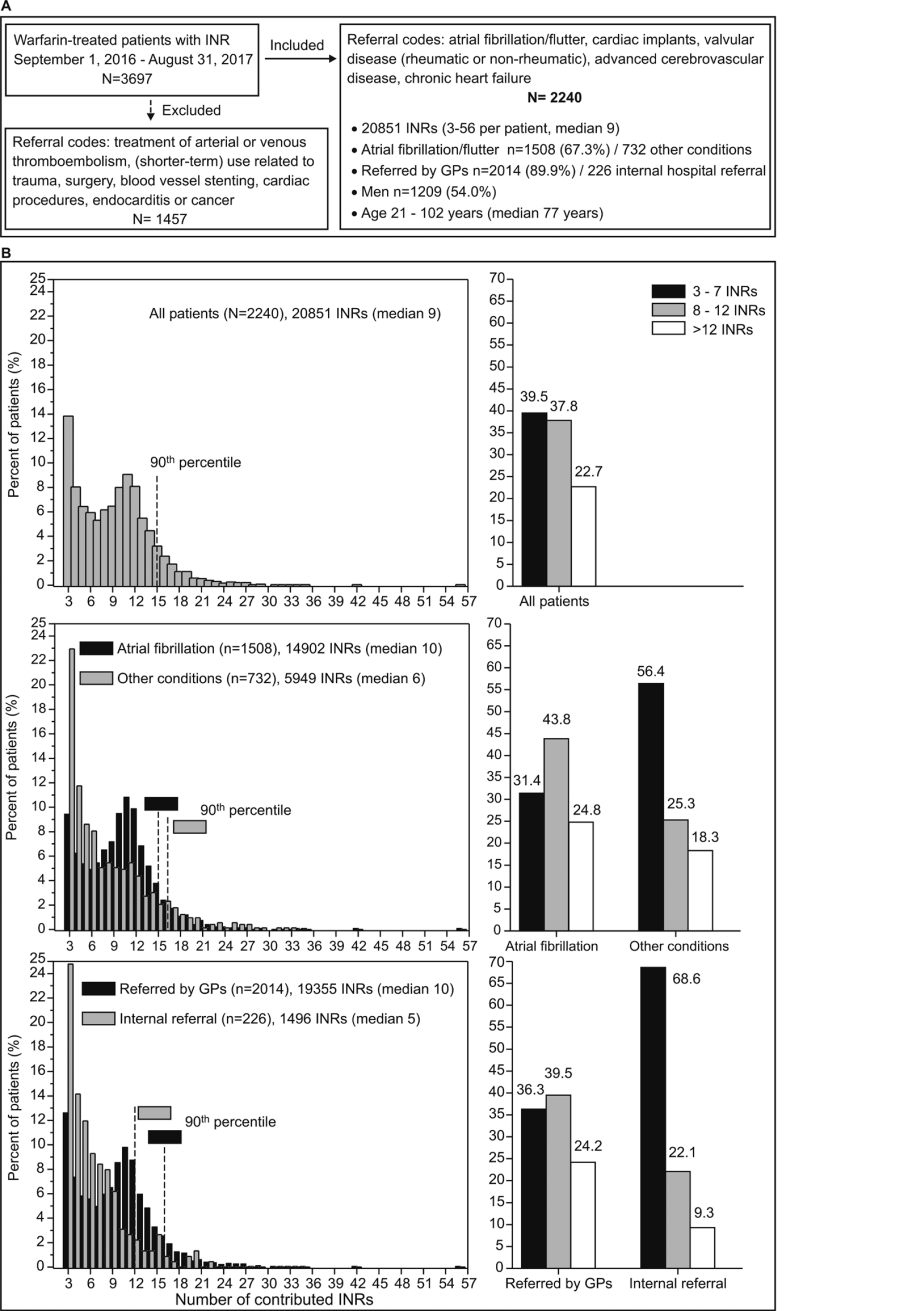
(**A**) Patient disposition. (**B**) Distribution of patients by the number of determined international normalized ratio values (INRs) – overall (upper row), by indication (middle row), and by referral mode (bottom row). GP – general practitioner.

The present analysis included 2240 patients with referral ICD codes suggesting an indication for long-term (life-long) thromboprophylaxis (Figure 1A): AF by far prevailed over other conditions – valvular disease, chronic heart failure with an indication for long-term thromboprohylaxis, cardiac implants, or a history of stroke or TIA (“advanced cerebrovascular disease”). Patients were predominantly referred by GPs (Figure 1A). Between 3 and 56 INRs were determined per patient (median 9), for 20 851 INRs (Figure 1A). More INRs per patient were determined in AF patients than in patients with other conditions, and in GP-referred patients than in internal hospital referrals (Figure 1B).

**Figure 2:**
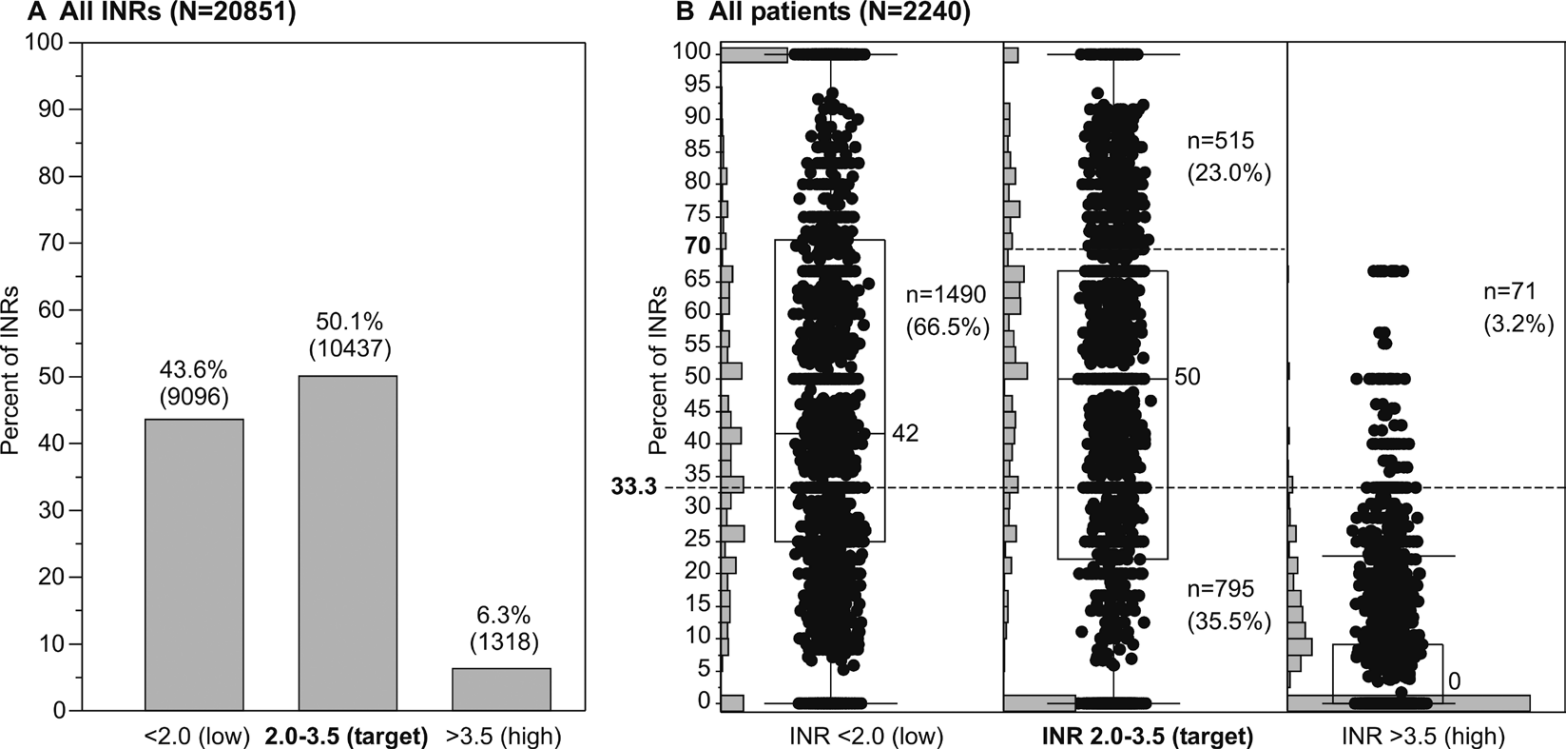
(**A**) Distribution of international normalized ratio values (INRs) below, within, or above the target range. (**B**) Raw individual INRs presented as the percentage of determined INRs that were below the target range (INR<2.0), within the target range (INR 2.0-3.5), and above it (INR>3.5). Dots are individual patient data, boxes indicate quartiles, horizontal lines indicate median values (depicted also numerically), and bars are inner fences. Data outside fences are outliers. Horizontal gray bars illustrate patient distribution in respect to percentage of low, target, and high INRs. Upper horizontal dashed line for “INR 2.0-3.5” indicates the cut-off of 70%: depicted is the number (percentage) of patients with ≥70% of the values within the target range, ie, those who were anticoagulated in line with the guidelines. Lower horizontal dashed line indicates the cut-off of 33.3%: depicted is the number (proportion) of patients with ≤33.3% of INRs within the target range, and the number (proportion) of patients with ≥33.3% of INRs that were too low or too high.

**Figure 3:**
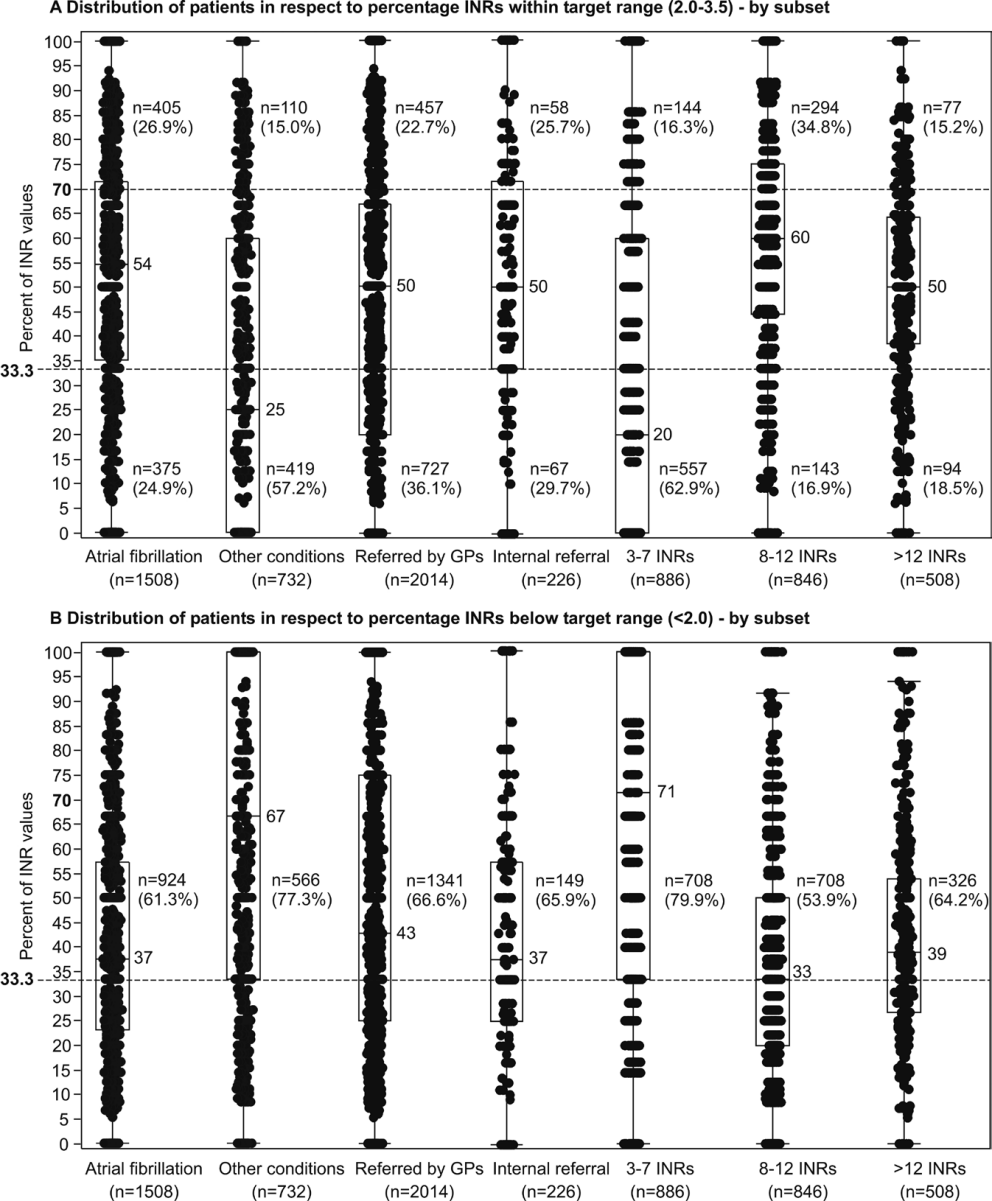
Distribution of patients by subsets based on indication, referral mode, and the number of determined INRs in respect to: (**A**) percentage of international normalized ratio values (INRs) within the target range; (**B**) percentage of INRs below the target range. Dots are individual patient data, boxes indicate quartiles, horizontal lines indicate median values (depicted also numerically), and bars are inner fences. Data outside fences are outliers. Horizontal dashed lines in (**A**) indicate cut-offs of 70% and 33.3% – depicted are numbers (proportions) of patients with ≥70% of INRs within the target range (adequately anticoagulated) and numbers (proportions) with ≤33.3% within the range. Horizontal dashed line in (**B**) indicates the cut-off of 33.3% – depicted are numbers (proportions) of patients with ≥33.3% INRs below the target range.

Only 50.1% of all INRs were within the target range, with a high proportion of INRs <2.0 and with only sporadic values >3.5 (Figure 2A). Crude proportions of INRs within the target range varied between 0 and 100% in individual patients (Figure 2B), and the median overall proportion was 50.0%. Only 23.0% of patients had ≥70% of the values in the target range (adequately anticoagulated), while 35.5% had ≤33.3% of the values in the target range (Figure 2B). At the same time, the proportions of INRs <2.0 also varied between 0 and 100%, and the median overall proportion was 42.0%, while 66.5% patients had ≥33.3% of the values <2.0 (Figure 2B). Therefore, values not in the target range were practically exclusively too low. Adequately anticoagulated patients (n = 515, 23.0%) and those who were not (n = 1725) were comparable regarding sex, age, and referral mode (Table 1), but in the former group there were more patients with AF (Table 1). Also, among adequately anticoagulated patients there were more patients with 8-12 INRs determined and fewer patients with 3-7 or >12 INRs determined (Table 1). The proportion of adequately anticoagulated patients was consistently low across all subsets (Figure 3A): in patients with AF (26.9%) and in those with other conditions (15.0%); in GP-referred (22.7%) and internally referred (25.7%) patients; and in patients with 3-7 INRs determined (16.3%) and those with >12 INRs determined (15.2%), likely reflecting oscillations at an earlier treatment stage or in patients with excessive variability, although it was somewhat higher in patients with 8-12 INRs determined (34.8%) (Figure 3A). At the same time, the proportion of patients with ≤33.3% of INRs in the target range varied from 16.9% to 62.9% (Figure 3A). Median proportions of values in the target range generally followed the pattern of the proportion of adequate anticoagulation (Figure 3A). Individual proportions of INRs <2.0 also varied between 0%-100% across the patient subsets, with medians mirroring those of proportions in the target range (Figure 3B). Accordingly, the proportion of patients with ≥33.3% of values <2.0 was consistently very high (53.9% to 72.7%) (Figure 3B).

**Table 1:** Patients characteristics, overall and by achieved recommended proportion (≥70%) of international normalized ratio (INR) values within the target range. Data are count (percent) or median (range)

	All	≥70% INRs in range	<70% INRs in range
N	2240	515	1725
Men	1209 (54.0)	267 (51.8)	942 (54.6)
Age	77 (21-102)	78 (40-97)	77 (21-102)
Referred by			
general practitioners	2014 (89.9)	457 (88.7)	1557 (90.3)
internal hospital referral	226 (10.1)	58 (11.3)	168 (9.7)
Main diagnosis			
atrial fibrillation/flutter	1508 (67.3)	405 (78.6)	1103 (63.9)
other conditions	732 (32.7)	110 (21.4)	622 (36.1)
Number of INRs determined	9 (3-56)	10 (3-25)	9 (3-56)
3-7	886 (39.6)	144 (28.0)	742 (43.0)
8-12	846 (37.8)	294 (57.1)	552 (32.0)
>12	508 (22.7)	77 (14.9)	431 (25.0)

**Figure 4:**
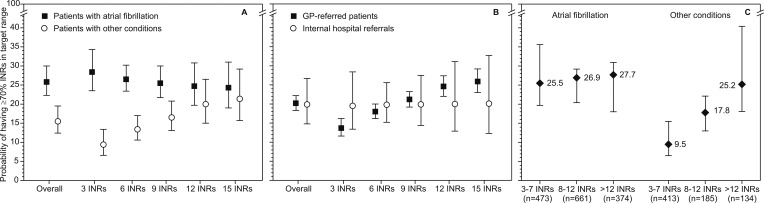
Adjusted probabilities (model in Table 2) of having ≥70% of INRs within the target range. (**A**) Adjusted probabilities (95% confidence intervals) from the interaction term between indication and the number of determined INRs. (**B**) Adjusted probabilities (95% confidence intervals) from the interaction term between referral mode and the number of determined INRs. (**C**) Adjusted probabilities summarized by by-indication-by category of numbers of determined INRs; diamonds are median probabilities (depicted numerically) and bars are ranges.

In a multivariate analysis (Table 2), the probability of adequate anticoagulation increased by 15% (95% CI -1 to 35%), with a 2-fold increase in the number of determined INRs. At the average number of INRs, the probability was by 67% higher in patients with AF than in those with other conditions (Table 2). A significant interaction between indication and the number of determined INRs with PR<1.0 indicated that the difference between the two subsets decreased with increasing number of INRs (Table 2). Conversely, in patients with AF, higher number of determined INRs was not associated with the probability of adequate anticoagulation, whereas in patients with other conditions it increased by 42% with a 2-fold increase in the number of INRs (Table 2, Figure 4A). Patients referred by GPs and those referred internally did not differ in the probability of adequate anticoagulation (Table 2), and the interaction between referral mode and the number of determined INRs suggested that the difference between subsets changed with increased number of determined INRs (Table 2). Conversely, in GP-referred patients, the probability increased by 32% with a 2-fold increase in number of INRs, while no such trend was observed in internally referred patients (Table 2, Figure 4B and 4C).

**Table 2:** Independent effects of diagnosis, referral mode, and the number of determined international normalized ratio (INR)* values on the probability of having ≥70% of INRs within the target range (INR 2.0-3.5): summary of multivariate analysis. Effects are prevalence ratios (PR) with 95% confidence intervals (CI). Prevalence ratios for the interaction terms indicate whether the effects of diagnosis and referral mode differed at different numbers of taken INR values, and *vice-versa*. Contrasts from the interaction terms are without *P*-values as they serve to illustrate differences in effects at different levels of the involved variables and not to test their significance

	PR (95% CI)	*P*
Number of INR values taken (by 2-fold)	1.15 (0.99-1.35)	0.074
Main diagnosis (atrial fibrillation/flutter vs other)	1.67 (1.36-2.04)	<0.001
Main diagnosis x number of INRs	0.66 (0.55-0.79)	<0.001
atrial fibrillation/flutter vs other - 3 INRs	3.00 (2.11-4.29)	—
atrial fibrillation/flutter vs other - 6 INRs	1.98 (1.58-2.48)	—
atrial fibrillation/flutter vs other - 9 INRs	1.55 (1.27-1.89)	—
atrial fibrillation/flutter vs other - 12 INRs	1.24 (0.99-1.54)	—
atrial fibrillation/flutter vs other - 15 INRs	1.14 (0.90-1.44)	—
number of INRs (by 2-fold) in patients with atrial fibrillation	0.94 (0.80-1.09)	—
number of INRs (by 2-fold) in patients with other conditions	1.42 (1.16-1.75)	—
Referred by (general practitioner [GP] vs internal hospital referral)	1.02 (0.76-1.37)	0.913
Referred by x number of INRs	0.77 (0.57-1.04)	0.085
referred by GPs vs internal referral - 3 INRs	0.70 (0.48-1.03)	—
referred by GPs vs internal referral - 6 INRs	0.91 (0.70-1.18)	—
referred by GPs vs internal referral - 9 INRs	1.06 (0.77-1.47)	—
referred by GPs vs internal referral - 12 INRs	1.23 (0.79-1.91)	—
referred by GPs vs internal referral - 15 INRs	1.29 (0.79-2.11)	—
number of INRs (by 2-fold) in GPs-referred patients	1.32 (1.20-1.44)	—
number of INRs (by 2-fold) in patients referred internally	1.01 (0.75-1.36)	—
Age (by 10 years)	1.03 (0.95-1.10)	0.434
Sex (men vs women)	0.96 (0.82-1.11)	0.568

*The number of determined INRs was ln-transformed and geometric-mean centered. The effect of the number of INRs is given by 2-fold increase {*PR = exp[β x ln(*2*)]*}.

## Discussion

The main finding of the present analysis is a very low proportion of patients with ≥70% INR values determined over a one-year period within the target range (INR 2.0-3.5). Since the proportion of INR values determined over a period of time that are within target range corresponds well with the TTR determined by the linear interpolation method (12), data strongly suggest that warfarin anticoagulation in the observed patients requiring life-long thromboprophylaxis was by far poorer than recommended (2,3). A potential limitation of our study might be the definition of the target INR range. However, it is highly unlikely that using the 2.0-3.0 range or different target ranges for prosthetic valve patients would have substantially changed the results since “off-target” INR values were almost exclusively <2.0. A further limitation might be that 39.5% of the included patients were evaluated based on a relatively low number of INRs (three to seven), which might have reflected earlier treatment stages (we did not identify newly treated patients) when INRs generally show greater variability. However, comparably low proportions of adequately anticoagulated patients were consistently observed among patients with 8-12 INR values (37.8% of all patients) and those with >12 INRs (22.7% of all patients). Moreover, in patients with AF (67.3% of all patients) a higher number of determined INRs was not associated with a higher probability of adequate anticoagulation, while in patients with other conditions the values increased from extremely low (9.5%) to very low (25.2%). In line with observations by other authors (9), the proportion of patients who were adequately anticoagulated was higher in patients with AF than in patients with other conditions requiring life-long thromboprohylaxis. This phenomenon has been attributed to more severe comorbidity and more extensive co-medication in patients with, eg, prosthetic valves, severe heart failure with atrial thrombi or very low ejection fraction, or those who suffered a stroke, which might make adequate anticoagulation more difficult to achieve (9). As expected, most of the “off-target” INRs were too low (INR<2.0) (across all levels of the number of determined INRs), a phenomenon that is commonly observed (12). It suggests that the prescribers might be more afraid of bleeding complications than of thromboembolic events, for which it is generally uncertain whether they will happen at all. Patients requiring life-long thromboprophylaxis commonly have comorbid conditions that *per se* increase the risk of major bleeding (eg, fatal or life-threatening by volume, intracerebral/intracranial), hence bleedings occur even in non-anticoagulated patients, and adequate anticoagulation does not increase this risk (1,17). On the other hand, inadequate anticoagulation, characterized by the predominance of low INR values, progressively increases the risk of thromboembolic events, which is considerably higher than the risk of major bleedings (1,12,17). While any form of thromboembolism can occur (either by an embolic mechanism or due to generally increased blood coagulability in these conditions), the most common is ischemic stroke (1-5). In a population-based study conducted in a Croatian county with a similarly sized and structured population as Zadar County (18), standardized incidence rate of first-ever ischemic stroke was considerably higher than reported in surrounding and developed European countries. Among incident patients, 36.1% suffered from AF and had additional risk factors for stroke that defined them as subjects who required anticoagulation, yet only 3.0% of them actually received regular anticoagulant treatment before the index event (18). During one-year follow-up, mortality was 3-fold lower in patients who were anticoagulated than in those who were not (adjusted hazard ratio 0.27, 95% CI 0.12-0.61) (19). In a subsequent case-control study in the same population (19), the occurrence of first-ever ischemic stroke was strongly associated with known modifiable risk factors, eg, smoking, unhealthy diet, uncontrolled hypertension, untreated dyslipidemia, AF and, particularly, no anticoagulation where it was indicated (19). In the light of these facts, the present data suggest that the high incidence of ischemic stroke and high case-fatality rates in stroke patients in Croatia are at least in part attributable to inadequate anticoagulation in patients in whom it is indicated.

In conclusion, we observed a highly inadequate level of warfarin anticoagulation in patients requiring life-long thromboprophylaxis in a Croatian county. Since the health care system organization is the same throughout the country, there is no reason to believe that the situation is relevantly different in any other part of Croatia. This is supported by the studies on the incidence and case-fatality rates of ischemic stroke in the Croatian population (18,19). Reports on the quality of anticoagulation with VKAs (and patient characteristics that may influence it) are extremely numerous and addressing them exceeds the scope of this work. The fact remains that even in the developed Western countries this quality may be less than desirable (20). Some of the cited studies (8-10) demonstrate that high quality is achievable, but this mainly depends on the organizational aspects. Therefore, although the present study provides no data that help explain the observations, it appears plausible that they are mainly due to the specifics of the health care system outlined in the Introduction. A recent survey on the quality of stroke prevention in atrial fibrillation in Croatia and the surrounding Balkan countries (facing similar health care organizational issues) (21) demonstrated that among 1198 atrial fibrillation patients treated with VKAs over at least previous 6 months, %TTR for the previous 3 months could be determined in only 224 (18.7%) due to the lack of data, indicating a poor practice of required continuous and structured monitoring. At the individual patient level, ESC guidelines suggest either NOACs or VKAs as the treatments of choice for patients with NVAF (the largest subset of patients requiring life-long anticoagulation), and preference of NOACs in NVAF patients in whom adequate anticoagulation with VKAs cannot be achieved (1). Given that major health care system reorganizations are not likely to happen in Croatia, NOACs appear to be a generally preferable choice for these patients. However, just as in the case of the surrounding countries (22), the use of NOACs in NVAF patients in Croatia is relatively limited (even if medically justified) due to a strong influence of the reimbursement policy.
